# Association of physical activity with cardiovascular and renal outcomes and quality of life in chronic kidney disease

**DOI:** 10.1371/journal.pone.0183642

**Published:** 2017-08-23

**Authors:** Yi-Chun Tsai, Hui-Mei Chen, Shih-Ming Hsiao, Cheng-Sheng Chen, Ming-Yen Lin, Yi-Wen Chiu, Shang-Jyh Hwang, Mei-Chuan Kuo

**Affiliations:** 1 Division of General Medicine, Department of Internal Medicine, Kaohsiung Medical University Hospital, Kaohsiung, Taiwan; 2 Division of Nephrology, Department of Internal Medicine, Kaohsiung Medical University Hospital, Kaohsiung, Taiwan; 3 Graduate of Medicine, Kaohsiung Medical University, Kaohsiung, Taiwan; 4 Department of Occupational Therapy, College of Health Sciences, Kaohsiung Medical University, Kaohsiung, Taiwan; 5 Department of Nursing, Kaohsiung Medical University Hospital, Kaohsiung, Taiwan; 6 Department of Psychiatry, Kaohsiung Medical University Hospital, Kaohsiung, Taiwan; 7 Faculty of Renal Care, Kaohsiung Medical University, Kaohsiung, Taiwan; Hospital Universitario de la Princesa, SPAIN

## Abstract

Patients with chronic kidney disease (CKD) are more readily prone to have impaired physical activity than the general population. The aim of this study is to examine the relationship between physical activity and adverse clinical outcomes and quality of life (QOL) in CKD. One hundred and sixty-one patients with CKD stages 1–5 was enrolled from February 2013 to September 2013 and followed up until June 2016. Physical activity was measured using high handgrip strength, 30-second chair stand, and 2-minute step. The QOL was assessed using the Taiwan version of the World Health Organization Quality of Life-BREF. Clinical outcomes included commencing dialysis, major adverse cardiovascular events (MACEs), and first hospitalization. Of all participants, 1 kgf increase in handgrip strength was significantly associated with 0.13 score increase in total scores of QOL and 0.05 score increase in physical domain of QOL in adjusted analysis. One time increase in 30-second chair stand was significantly correlated with 0.14 score increase in psychological domain of QOL. Over a mean follow-up period of 29.1±11.2 months, 37 (23.0%) reached commencing dialysis, 11(6.8%) had MACEs, and 50(31.1%) had first hospitalization. High handgrip strength (hazard ratio (HR): 0.89, 95% CI: 0.84–0.96) and high 2-minute step (HR: 0.04, 95% CI: 0.01–0.95) were significantly associated with decreased risk for commencing dialysis in multivariate analysis. Thirty-second chair-stand was negatively associated with MACEs (HR: 0.65, 95%CI: 0.47–0.89) and first hospitalization (HR: 0.84, 95%CI: 0.74–0.95). In conclusion, physical activity is a potential predictor of QOL and adverse clinical outcomes in patients with CKD.

## Introduction

Patient with chronic kidney disease (CKD) are more readily prone to have impaired physical activity than the general population [1]. CKD might result in accumulation of metabolic waste products and hormone disturbances, further contributing to skeletal muscle dysfunction, which is associated with physical performance [2]. Impaired physical activity affects functional and psychosocial status, as well as quality of life (QOL). Chronic health problems and weak psychosocial and occupation roles might cause deterioration of well-being [3]. The correlation between QOL and physical activity measured by muscle or cardiorespiratory endurance is limited in CKD population.

Reduced physical function has been considered as a predictor of cardiovascular morbidity and all-cause mortality in normal individuals [4,5]. Accumulating evidences indicates that poor physical activity is associated with mortality in CKD patients whether on dialysis or not [1,6,7]. However, there is no analysis of the association of physical activity with major adverse cardiovascular events (MACEs) in CKD cohort. Besides, few reports have analyzed the relationship between physical activity and poor renal progression [7,8]. Low handgrip strength and less walking habit defined by questionnaires are correlated with increased risk for commencing dialysis [7,8]. Regrettably, handgrip strength only presents as upper extremity endurance, a part of whole body physical activity, and there is some limitation of accurate physical activity as assessed by self-record questionnaire.

Accordingly, we hypothesized that impaired physical activity is associated with adverse clinical outcomes in patients with CKD not on dialysis. The study attempts to determine the relationship between physical activities such as the 2-minute step, handgrip strength and the 30-second chair-stand tasks and QOL and adverse clinical outcomes including commencing dialysis, MACE, and all-cause hospitalization in patients with CKD stages 1–5.

## Materials and methods

### Study participants

This prospective study enrolled 172 CKD stages 1–5 patients in our integrated CKD program for more than 3 months from February 2013 to September 2013 at one hospital in Southern Taiwan as our previous study [9]. Patients with instability, maintenance dialysis, and shunt implantation, New York Heart Association (NYHA) class III~IV congestive heart failure, and obstructive lung disease were excluded in this study. We also exclude 11 patients with losing follow-up during observation period. Finally, 161 patients entered further analysis. The study protocol was approved by the Institutional Review Board of the Kaohsiung Medical University Hospital (KMUH-IRB- 20120203). CKD was staged based on K/DOQI definitions and the estimated glomerular filtration rate (eGFR) was calculated using the equation of the 4-variable Modification of Diet in Renal Disease (MDRD) Study [10]. Informed consents were obtained in written form from patients and all clinical investigations were conducted according to the principles expressed in the Declaration of Helsinki.

### Assessment of physical activity

Physical activity was determined using both the questionnaire and indices. The questionnaire (S1 and S2 Files.) used in this study was based on part of National Health and Nutrition Examination Survey (NHANES) physical activity questionnaire (https://www.cdc.gov/nchs/nhanes/nhanes_questionnaires.htm) [11]. Beddhu et al. had utilized this questionnaire in CKD patients to analyze the association between physical activity and all-cause mortality [1]. The activity was defined as level of Metabolic Equivalent of Task (MET) that was scored and categorized into three groups, including low activity (MET score less than 3, walking slowly: 2.3), middle activity (MET score of 3–6, walking fast: 3.6 and riding a bicycle: 5.5), and high activity (MET score above 6, running:7.0 and exercise biking: 10.0) [12]. Indices of physical activity, such as the 2-minute step test, handgrip, and the 30-second chair-stand test were performed at enrollment of this study. Cardiorespiratory endurance was evaluated using the 2-minute step test, while CKD patients alternately raised each knee to the midway between iliac crest and the patella for 2 minutes and the number of repetitions were recorded [13,14]. Grip strength dynamometer (T.K.K. 5401 GRIP-D, Takei scientific instruments Co., LTD, Japan) was used to measure handgrip strength (kgf) as an indicator of upper extremity muscle endurance. Two consecutive measurements were performed at intermediate forearm position with the elbow flexed at 90 degree, while patients were in a seated position. The mean of the maximal values for each hand was used for analysis [15]. Lower extremity muscle endurance was measured using the 30-second chair-stand test that the number of times raised to a full stand in 30 seconds in each patient was used for analysis [16].

### Measurement of quality of life (QOL)

The QOL of CKD patients was assessed using the Taiwan version of the World Health Organization Quality of Life-BREF (WHOQOL-BREF) [17], which included the total scores and four health domains, including physical, psychological, social relations and environment domains. Patients answered the questionnaires with the aid of explanations from trained nurses at enrollment.

### Data collection

Demographic and clinical information of patients was obtained from interviews and medical records at enrollment. Blood and urine samples were obtained at the same time of physical activity measurement. Patients were asked to fast for at least 12 hours before blood sample collection for the biochemistry study. The severity of proteinuria was determined using urine protein-creatinine ratio (PCR). Blood pressure was recorded as the mean of two consecutive measurements with 5-minute intervals, using one single calibrated device. Hypertension, diabetes, hyperlipidemia and gout were defined as those with a medical history through chart review. Cardiovascular disease was defined as a history of acute or chronic ischemic heart disease, heart failure, or myocardial infarction. Information of medications including β-blocker, calcium channel blocker, angiotensin converting enzyme inhibitor (ACEI), and angiotensin II receptor blocker (ARB) before and after enrollment was obtained from medical records.

### Clinical outcomes

Patients were contacted at outpatient clinics at 3-month intervals to ascertain the clinical status. Clinical outcomes included commencing dialysis, MACE and first hospitalization. Commencing dialysis was defined as either requiring maintenance hemodialysis or peritoneal dialysis and confirmed by reviewing medical charts or catastrophic illness certificate (issued by the Bureau of National Health Insurance in Taiwan). MACE was defined as new onset of acute myocardial infarction, acute hemorrhagic or ischemic stroke, and hospitalization related to acute phase of congestive heart failure or arrhythmia. Hospitalization was defined as the first hospitalization not admitted for vascular access creation. Patients were censored at death, the last contact, or the end of observation on 30 June 2016.

### Statistical analysis

Continuous variables were expressed as mean±SD or median (25^th^, 75^th^ percentile), as appropriate, and categorical variables were expressed as percentages. Skewed distribution continuous variables were log-transformed to attain normal distribution. The significance of differences in continuous variables between groups was tested using independent t-test or the Mann-Whitney U analysis as appropriate. The difference in the distribution of categorical variables was tested using the Chi-square test. Spearman correlation and linear regression models were utilized to evaluate the association of physical activity with quality of life in CKD patients. All the variables in Table 1 tested by univariate analysis and those variables with P-value less than 0.05, plus age, sex, and serum creatinine were selected in multivariate linear regression analysis. Time-to-event survival analysis was used to analyze physical function as a predictor of the risk of clinical outcomes. Cox regression models were utilized to examine the risks for clinical outcomes. Traditional risk factors including age, sex, diabetes mellitus, cardiovascular disease, ACEI/ARB usage, urine PCR cut 1mg/mg, and serum creatinine, albumin and hemoglobin were selected for multivariate cox regression analysis. Furthermore, patients were stratified by age, sex, serum albumin levels to investigate the effect of interaction of well-known risk factors on the association between physical activity and clinical outcomes in subgroup analysis. Statistical analyses were conducted using SPSS 18.0 for Windows (SPSS Inc., Chicago, Illinois) and the graphs were made by GraphPad Prism 5.0 (GraphPad Software Inc., San Diego CA, USA). Statistical significance was set at a two-sided p-value of less than 0.05.

**Table 1:** The clinical characteristics of study subjects stratified by CKD stages.

	Entire Cohort (n = 161)	CKD stage 1–3 (n = 65)	CKD stage 4–5 (n = 96)	P-value
Demographic variables				
Age, year	67.2±7.8	67.7±7.8	66.8±7.8	0.48
Sex (male), n%	87(54.0)	38(58.5)	49(51.0)	0.35
Smoke (yes), n%	46(28.6)	19(29.2)	27(28.1)	0.87
Alcohol (yes), n%	29(18.0)	12(18.5)	17(17.7)	0.90
Marriage (yes), n%	132(82.0)	53(81.5)	79(82.3)	0.90
Religion (yes), n%	135(90.0)	53(86.9)	82(92.1)	0.29
Occupation (yes), n%	50(31.1)	17(26.2)	33(34.4)	0.26
Education				
(senior high school or above)	83(51.6)	38(58.5)	45(46.9)	0.15
Cardiovascular disease (yes), n%	24(15.0)	6(9.2)	18(18.9)	0.09
Diabetes (yes), n%	60(37.3)	18(27.7)	42(43.8)	0.04
Hypertension (yes), n%	114(70.8)	37(56.9)	77(80.2)	0.001
Hyperlipidemia (yes), n%	66(41.0)	20(30.8)	46(47.9)	0.03
Gout (yes), n%	28(17.4)	10(15.4)	18(18.8)	0.58
Body Mass Index, kg/m^2^	24.4±3.8	24.4±4.0	24.3±3.8	0.82
Physical activity				
2-minute step	105(92,115)	106(94,120)	104(90,112)	0.16
Handgrip strength, kgf	26.9±7.7	28.0±7.5	26.1±7.8	0.12
30-second chair stand	11.2±3.1	11.9±3.5	10.7±2.8	0.02
Questionnaires of exercise capacity				0.005
No exercise (yes), n%	8(5.0)	2(3.1)	6(6.3)	
Low activity (yes), n%	113(70.2)	45(69.2)	68(70.8)	
Middle+High activity (yes), n%	40(24.8)	21(32.3)	19(19.8)	
Medications				
β-blocker (yes), n%	28(17.4)	9(13.8)	19(19.8)	0.32
Calcium channel blocker (yes), n%	69(42.9)	15(23.1)	54(56.3)	<0.001
ACEI/ARB (yes), n%	52(32.3)	10(15.4)	42(43.8)	<0.001
Lipitor (yes), n%	33(20.5)	10(15.4)	23(24.0)	0.18
Quality of life				
Total score	52.3±5.3	53.2±5.2	51.4±5.2	0.03
Physical domain	11.9±1.5	12.1±1.5	11.7±1.5	0.14
Psychological domain	12.6±1.7	12.8±1.7	12.3±1.7	0.05
Social domain	13.2±2.0	13.5±2.1	12.9±2.0	0.05
Environment domain	14.7±1.5	14.8±1.5	14.6±1.4	0.25
Laboratory parameters				
Blood urea nitrogen, mg/dl	33.5(21.4,55.3)	19.7(14.7,23.8)	48.4(34.2,65.3)	<0.001
Creatinine, mg/dl	3.1±2.4	1.2±0.5	4.3±2.3	<0.001
eGFR, ml/min/1.73m^2^	34.5±28.8	62.2±26.1	15.7±7.7	<0.001
Hemoglobin, g/dl	11.0±2.0	12.5±1.8	10.5±1.8	<0.001
Albumin, g/dl	4.2±0.3	4.3±0.4	4.2±0.4	0.18
Phosphate, mg/dl	4.1(3.5,4.6)	3.6(3.2,3.9)	4.3(3.9,4.8)	<0.001
Calcium, meq/L	9.1±0.6	9.3±0.4	9.0±0.6	0.03
Sodium, meq/L	139.0±2.6	139.7±2.0	138.6±2.8	0.01
Potassium, meq/L	4.2±0.5	4.1±0.4	4.3±0.5	0.001
Cholesterol, mg/dl	185.9±35.6	186.5±32.7	185.4±37.7	0.84
Triglyceride, mg/dl	117.0(82.0,159.0)	115.1(81.0,144.5)	120.0(81.5,167.0)	0.23
Urine protein-creatinine ratio, g/g	0.8(0.3,1.6)	0.2(0.1,0.5)	1.0(0.5,1.7)	<0.001

*Notes*: Data are expressed as number (percentage) for categorical variables and mean±SD or median (25^th^, 75^th^ percentile) for continuous variables, as appropriate.

Abbreviations: CKD, chronic kidney disease; eGFR, estimated glomerular filtration rate

## Results and discussion

### Characteristics of entire cohort

The mean age of study patient was 67.2±7.8 years (from 52 to 85 years) and 87(54%) patients were male (Table 1). The mean eGFR was 34.5±28.8 ml/min/1.73m^2^ and 65(40.4%) had CKD stages 1–3 and 96(59.6%) had CKD stages 4–5. Among 161 patients, 70.8% were hypertensive, 37.3% were diabetic and 15.0% had cardiovascular disease. The proportion of diabetes, hypertension, hyperlipidemia, and usage of Calcium channel blocker and ACEI/ARB was higher in patients with CKD stages 4–5 than in those with CKD stages 1–3. Patients with CKD stages 4–5 had higher serum phosphate, potassium and urine PCR than those with CKD stages 1–3. Lower hemoglobin, and serum calcium and sodium were found in patients with CKD stages 4–5 than in those with CKD stages 1–3.

### Physical function of entire cohort

Of all patients, 8(5.0%) had no exercise capacity, 113(70.2%) had low exercise capacity, and 40(24.8%) had middle or high exercise capacity (Table 1). The median of the 2-minute step was 105 (92,115). The means of handgrip strength and the 30-second chair stand were 26.9±7.7 kgf and 11.2±3.1 respectively. Patients with CKD stages 4–5 had lower number of the time of 30-second chair stand than those with CKD stages 1-3(10.7±2.8 *v*.*s*.11.9±3.5, P = 0.02). There was no significant difference of the 2-minute step and handgrip strength between the two groups.

### Physical activity and quality of life (QOL)

The mean of the total scores of QOL was 52.3±5.3 in patients with CKD stages 1–5. Patients with CKD stages 1–3 had higher total scores of QOL than those with CKD stages 4–5 (53.2±5.2 *v*.*s*. 51.4±5.1, P = 0.03, Table 1). In univariate linear regression, handgrip strength was positively correlated with all domains and total scores of QOL except for psychological domain. A positive relationship was found between the 30-second chair stand and all domains and total scores of QOL, except environment domain (Table 2). In multivariate linear analysis of the correlation between physical activity and QOL after adjustment of age, sex, serum creatinine, and all variables of P<0.05 in univariate analysis, 1 kgf increase in handgrip strength was significantly associated with 0.13 score increase in total scores of QOL (95% confidence index (CI): 0.01–0.24) and 0.05 score increase in physical domain of QOL (95% CI: 0.02–0.08). One time increase in the 30-second chair stand was significantly correlated with 0.14 score increase in psychological domain of QOL (95% CI: 0.05–0.24).

**Table 2:** Linear regression analysis of association of physical activity with quality of life in patients with chronic kidney disease.

	2-minute step*		Handgrip strength, kgf		30-second chair-stand	
	Univariate β(95%Cl)	multivariate β(95%Cl)	Univariate β(95%Cl)	multivariate β(95%Cl)	Univariate β(95%Cl)	multivariate β(95%Cl)
Total scores	7.08(-0.65,14.81)	-0.39(-9.33,8.56)	0.17(0.06,0.27)^a^	0.13(0.01,0.24)^b^	0.30(0.04,0.55)^b^	0.04(-0.05,0.13)
Physical domain	2.21(-0.03,4.46)	0.68(-1.89,3.24)	0.05(0.02,0.08)^a^	0.05(0.02–0.08)^a^	0.08(0.01,0.15)^b^	0.04(-0.05,0.13)
Psychological domain	1.76(-0.80,4.32)	0.56(-2.60,3.72)	0.03(-0.00,0.06)	0.04(-0.02,0.09)	0.13(0.04,0.21)^a^	0.14(0.05,0.24)^a^
Social domain	1.83(-1.17,4.85)	0.51(-2.84,3.86)	0.05(0.01,0.08)^b^	0.04(-0.03,0.09)	0.11(0.01,0.21)^b^	0.07(-0.04,0.18)
Environmental domain	1.26(-0.87,3.40)	-0.01(-2.43,2.41)	0.04(0.01,0.06)^b^	0.04(-0.02,0.10)	-0.02(-0.09,0.05)	-0.05(-0.13,0.03)

Multivariate linear regression analysis was adjusted for all variables of p-value<0.05 plus age, sex, and serum creatinine level

*Log-formed 2-minute step

^a^P<0.01

^b^P<0.05

### Physical activity and clinical outcomes

Of 161 patients, 37(23.0%) reached commencing dialysis, 11(6.8%) had MACE, and 50(31.1%) had first hospitalization over a mean follow-up period of 29.1±11.2 months (Table 3). Eleven (6.8%) patients were lost to follow-up. No death was recorded during the follow-up period. The causes of MACE included four of acute myocardial infarction, four of acute congestive heart failure, one of brain infarction, and two of arrhythmia. Infection accounted for 28% of hospitalizations, MACE for 22%, malignancy for 14%, renal function progression for 14%, gastrointestinal bleeding for 8%, bone fracture due to accident for 8%, and other causes (head injury, impaired memory, and hypoglycemia) for 6%.

**Table 3:** Clinical outcomes of CKD patients stratified by CKD stages.

	Entire cohort (n = 161)	CKD stage 1–3 (n = 65)	CKD stage 4–5 (n = 96)	P-value
Follow-up period, months	29.1±11.2	31.4±8.6	27.5±12.4	0.02
Commencing dialysis, n(%)	37(23.0)	1(1.5)	36(37.5)	<0.001
Major adverse Cardiovascular events, n(%)	11(6.8)	1(1.5)	10(10.4)	0.02
All-causes hospitalization, n(%)	50(31.1)	17(26.2)	33(34.4)	0.26

Data are expressed as number (percentage) for categorical variables and mean±SD or median (25^th^, 75^th^ percentile) for continuous variables, as appropriate.

**Fig 1:**
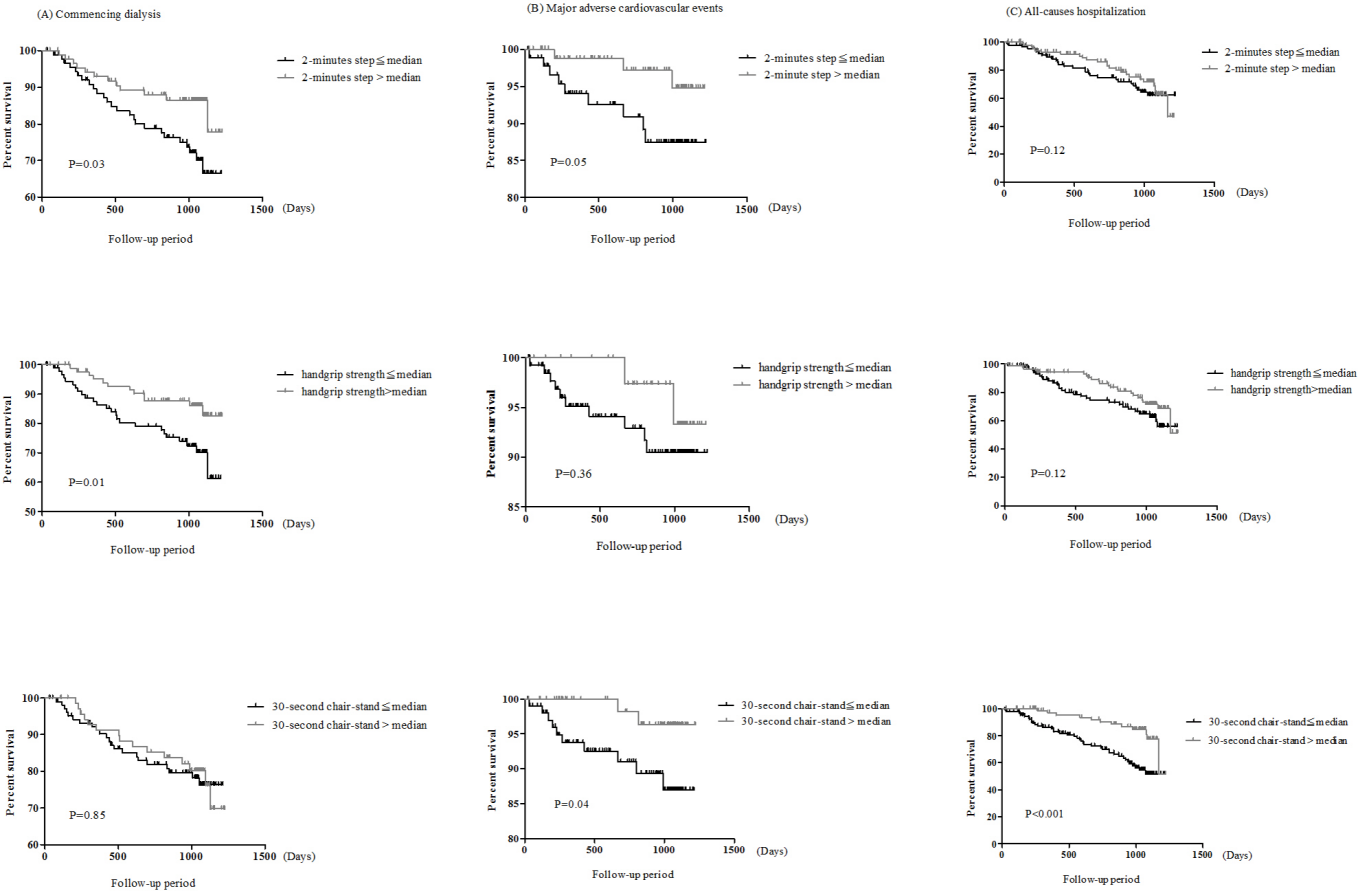
**The cumulative probability of (A) commencing dialysis (B) major adverse cardiovascular events (MACE) (C) first hospitalization according to physical activity.** The median of 2-minute step, handgrip strength, and 30-second chair stand was 105, 26.4kgf, and 11 respectively.

Kaplan-Meier survival curve showed a significant relationship between handgrip strength and the 2-minute step and commencing dialysis, and the 30-second chair-stand was associated with both MACE and all-causes hospitalization (Fig 1). High handgrip strength (hazard ratio (HR): 0.89, 95% CI: 0.84–0.96, P = 0.005) and high 2-minute step (HR: 0.04, 95% CI: 0.01–0.95, P = 0.04, Table 4) were significantly associated with decreased risk for commencing dialysis in multivariate cox regression analysis. One increase of the time of 30-second chair-stand was significantly associated with decreases of 35% risk for MACEs (HR: 0.65, 95%CI: 0.47–0.89) and 16% risk for first hospitalization (HR: 0.84, 95%CI: 0.74–0.95). There was no significant association of exercise activity measured by questionnaires with commencing dialysis, MACEs or first hospitalization.

**Table 4:** The adjusted risks for commencing dialysis, major adverse cardiovascular events (MACEs), and all causes hospitalization according to physical function.

	Commencing dialysis		MACEs		All causes hospitalization	
	Hazard ratio (95% Cl)	P-value	Hazard ratio (95% Cl)	P-value	Hazard ratio (95% Cl)	P-value
2-minute step	0.04(0.01–0.95)	0.04	0.04(0.00–30.05)	0.33	0.94(0.04–22.51)	0.97
handgrip strength, kgf	0.89(0.84–0.96)	0.005	0.99 (0.87–1.13)	0.94	0.96(0.90–1.02)	0.17
30-second chair-stand	1.02(0.88–1.17)	0.82	0.65(0.47–0.89)	0.008	0.84(0.74–0.95)	0.01

Abbreviations: CI, Confidence Interval

Adjusted model: age, sex, cardiovascular disease, diabetes mellitus, angiotensin converting enzyme inhibitor/angiotensin II receptor blocker usage, urine protein-creatinine ratio cut at 1mg/mg, and serum creatinine, albumin and hemoglobin level

**Fig 2:**
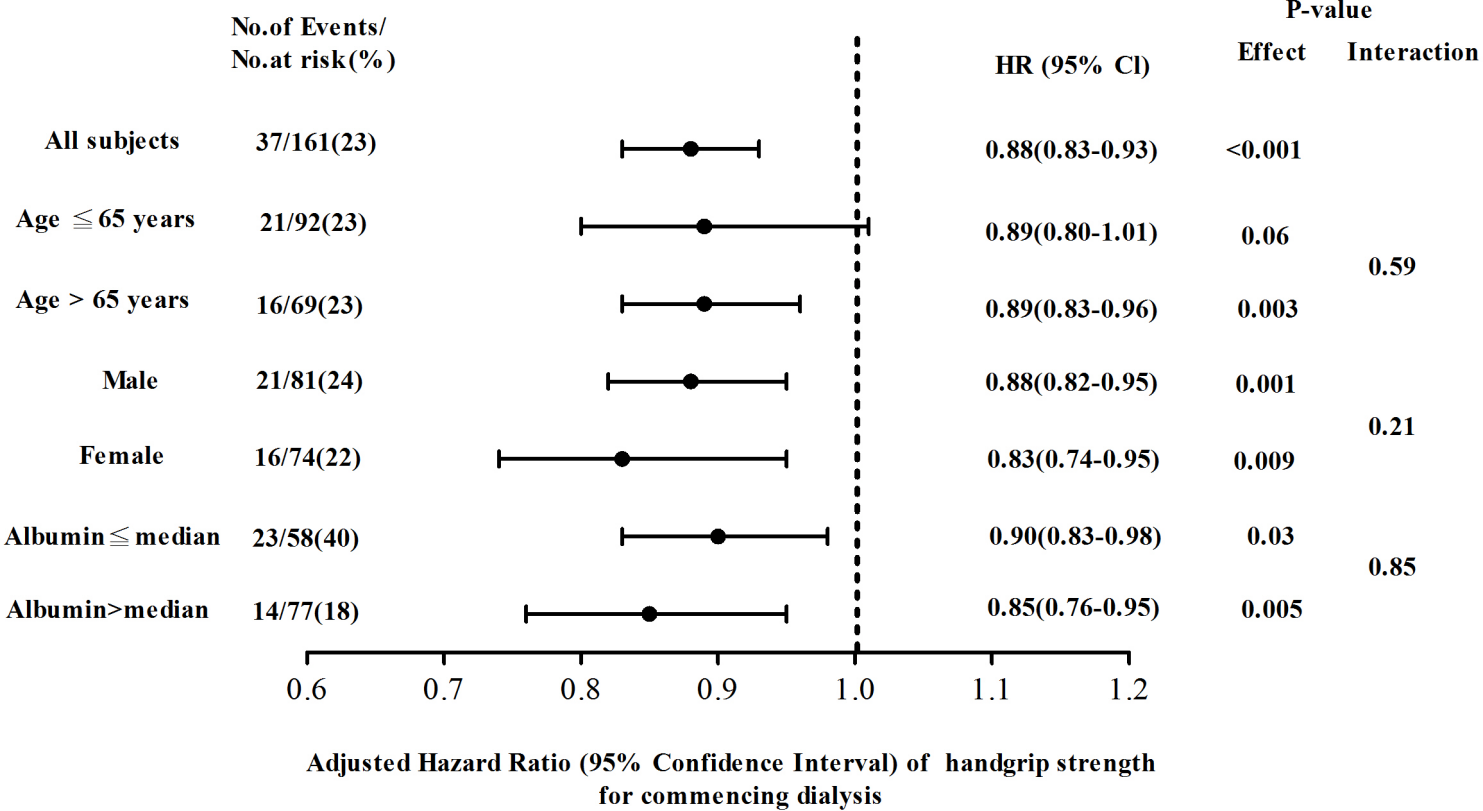
Adjusted hazard ratios (HRs) of commencing dialysis for handgrip strength in all chronic kidney disease patients stratified by age, sex, and serum albumin level. Ratios were adjusted for age, sex, creatinine. The median of serum albumin was 4.2 g/dl. 95% CI, 95% confidence intervals.

Further subgroup analysis found the decrease in handgrip strength was significantly associated with increased risk for commencing dialysis independent of sex and serum albumin level (Fig 2). However, this correlation between handgrip strength and commencing dialysis was not consistent in CKD patients with age of 65 years or less. There was no significant interaction between subgroups.

## Discussion

This study investigated the relationship between physical activity and adverse clinical outcomes in patients with CKD stages 1–5 not on dialysis. The results showed a positive correlation between handgrip strength and the score of physical domain and total scores of QOL and the increase in 30-second chair-stand was correlated with high scores of psychological domain of QOL. High handgrip strength and the 2-minute step were significantly associated with decreased risk for commencing dialysis. The negative association of the 30-second chair-stand with MACEs and first hospitalization was mentioned in CKD patients after adjustment of well-known risk factors.

The novel result of this study revealed that poor lower extremity endurance was associated with increased risks for MACEs and first hospitalization. To our best knowledge, this is the first report in CKD patients with this finding. Lower extremity activity is complex and depends on muscle strength, agility, balance and neuromuscular control [18]. Lower extremity activity presents integral physical activity. Newman et al. supported the notion that inability to complete long-distance corridor walk was correlated with a higher risk for cardiovascular mortality in community-dwelling older adults [5]. Roshanravan et al. enrolled patients with CKD stages 2–4 and demonstrated that impaired physical performance of the lower extremities, such as slow gait speed, was a strong predictor of mortality [6]. Additionally, impaired lower extremity performance has been known as a predictor of hospitalization in general elders [19]. In our CKD patients, there was no significant difference of demographic characteristics, malnutrition-inflammation, such as serum albumin, and baseline renal function between those reaching MACEs and those not. Patients reaching MACEs had higher proportion of diabetes and heart disease history, as traditional cardiovascular risk factors, than those not reaching MACEs. Nevertheless, we adjusted these cardiovascular risk factors and the relationship between the 30-second chair-stand and MACEs and first hospitalization was still accordant. Lower extremity endurance is a potential predictor of MACEs and first hospitalization in CKD cohort.

The other novel finding is that higher upper extremity endurance was correlated with not only greater physical domain but also total scores of QOL in our CKD patients not on dialysis. We also found a positive correlation between lower extremity endurance and psychological domain of QOL. There was no association of cardiorespiratory endurance with QOL. Conversely, Lopes et al. reported that aerobic activity, but not strength/flexibility activity measured by questionnaires, was associated positively with QOL in patients on maintenance dialysis [18]. The inconsistent results might be related to different assessment methods of physical activity and distinct populations in the two studies. Handgrip strength had been considered as an indicator of overall QOL in community-dwelling older adults [20]. As normal individuals, this correlation was also mentioned in CKD cohort, and furthermore, high handgrip strength was associated with better performance of physical domain of QOL after adjustment of age, sex, baseline renal function and affecting factors of QOL. Besides, the correlation between the 30-second chair-stand and psychological domain, not social domain of QOL was shown in CKD patients, while it was unlike that of community-dwelling older adults [20]. It is difficult to differentiate the line between physiological domain and social domain because of complicated interaction between them. Impaired lower extremity endurance might be related to depression symptoms, thereby making CKD patients have no courage to create interpersonal relationships. Therefore, physical activity is one of the major associated components of QOL in CKD patients.

We found that the decreased 2-minute step, as an indicator of cardiopulmonary endurance, was associated with increased risk for commencing dialysis. Besides, a negative correlation between handgrip strength and commencing dialysis in CKD patients, as for the study conducted by Chang et al. [8]. However, the mechanism mediating commencing dialysis by physical activity has not been well understood. CKD itself is a process of inflammation-malnutrition, and chronic inflammation-malnutrition has played a role in poor renal function progression [21]. Inflammation would cause protein-energy wasting, malnutrition, and atherosclerosis [22]. Thus, we stratified CKD patients by age, sex, and serum albumin level, and the significant association of handgrip strength with commencing dialysis was consistent independent of sex and serum albumin level. Nevertheless, there is a complicated interaction between physical activity and inflammation-malnutrition. Further study is needed to investigate the interactional effect of physical activity and inflammation-malnutrition on poor renal progression.

Physical activity has been thought to be a risk factor for development of CKD [23]. Maximal exercise tolerance might be affected in the early stage of CKD [24]. Regular physical activity is associated with anti-inflammatory effect [25]. Physical activity could increase nitric oxide, lessen reactive oxygen species, and improve vascular insulin signaling, thereby ameliorating cardiovascular endothelial function [26]. Regular physical activity might increase self-confidence, reduce depression symptoms, and have opportunities of contacting social environment [27]. With regard to the predictability of adverse outcomes, habitual physical activity probably improves physical and psychological domains of QOL, furthering clinical performance. Since most participants only did light physical activity, the outcome was significantly associated with decreased risks for commencing dialysis or MACEs in the present study. Even doing light physical activity, it can reduce the risk for adverse outcome and improve or maintain the level of QOL. Therefore, physicians should be aware of particular conditions associated with impaired physical activity and might target rehabilitation programs in patients with CKD.

Some limitations of the present study must be considered. Firstly, the sample size and the event number of MACEs of our study were relative small. It is difficult to performed subgroup analysis to investigate the influence of interaction between the 30-second chair-stand and associated variables on increased risks of MACEs. Besides, relatively small numbers of study participants might affect the predictability of renal outcomes. The HR of handgrip strength for commencing dialysis had marginal significance (P = 0.06) in CKD patients with age of 65 years or less, where adjusted HR was similar in the subgroup of age above 65years. Thus, this data suggests acceptable statistic power and handgrip strength might be a risk factor for poor renal progression independent of age. In addition, physical function was measured once at enrollment. The influence of time-varying physical function on adverse clinical outcomes could not be estimated. Finally, some participants, who received shunt creation and/or were unable to independently ambulate, were excluded in this study. These results could not be therefore generalized to all CKD patients not on dialysis.

## Conclusions

In conclusion, upper and lower extremity endurance was positively correlated with QOL in CKD patients. Impaired lower extremity endurance was significantly associated with increased risks for MACEs and first hospitalization, and the relationship between poor upper extremity endurance and entering commencing dialysis was mentioned. Impaired physical activity might be a potential predictor of adverse clinical outcomes in CKD. Further studies are needed to evaluate the effect of trained exercise program on improvement of clinical outcomes in CKD patients.

## Supporting information

S1 File National Health and Nutrition Examination Survey (NHANES) physical activity questionnaire. (PDF)

S2 File Chinese version of physical activity questionnaire. (PDF)
