# Supplementary material for: Association of physical activity with cardiovascular and renal outcomes and quality of life in chronic kidney disease
Source: PLoS One. 2017 Aug 23;12(8):e0183642. doi: 10.1371/journal.pone.0183642 (PMC5568323; doi:10.1371/journal.pone.0183642)
Supplement: S1 File — (PDF) [file pone.0183642.s001.pdf]

Supplementary Table 1: Questions for adults on physical activity with corresponding variable names, NHANES 2003-2006

Domain	Questions	2003-2004	2005-2006
Walking and biking	Over the past 30 days, have you walked or bicycled as part of getting to and from work, or school, or to do errands? yes or no or unable to do activity	PAD020	PAQ020
	(IF YES) How often did you do this? enter number of days, weeks, or months	PAQ050Q PAQ050U	PAQ050
	(IF YES) On those days when you walked or bicycled, about how long did you spend altogether doing this? enter number of minutes or hours	PAD080	PAD080
Home/yard physical activity (# minutes per 30 days)	Over the past 30 days, did you do any tasks in or around your home or yard for at least 10 minutes that required moderate or greater physical effort? By moderate physical effort, I mean tasks that caused light sweating or a slight to moderate increase in your heart rate or breathing, such as raking leaves, mowing the lawn, or heavy cleaning.	PAQ100	PAQ100
	(IF YES) How often did you do these tasks in or around your home or yard for at least 10 minutes at a time that required moderate or greater physical effort? enter number of times (past 30 days)	PAD120	PAD120
	(IF YES) About how long did you do these tasks each time? enter number of minutes	PAD160	PAD160
General description of daily activities	Please tell me which of the sentences best describes your usual daily activities? (1) You sit during the day and do not walk about very much. (2) You stand or walk about quite a lot during the day but do not have to carry or lift things very often. (3) You lift or carry light lads or have to climb stairs or hills often. (4) You do heavy work or carry heavy loads.	PAQ180	PAQ180
Vigorous physical activity (PADLEVEL=2) (# minutes per 30 days)	Over the past 30 days, did you do any vigorous activities for at least 10 minutes that cause heavy sweating, or large increases in breathing or heart rate? Some examples are running, lap swimming, aerobics classes, or fast bicycling. (yes or no or unable to do activity)	PAD200	PAD200
	(IF YES) What vigorous activities did you do? code all activities	PADACTIV	PADACTIV
	(IF YES) How often did you do X activity? enter number (past 30 days)	PADTIMES	PADTIMES
	(IF YES) On average, about how long did you do X each time? enter number of minutes	PADDURAT	PADDURAT
Moderate physical activity (PADLEVEL=1) (# minutes per 30 days)	Over the past 30 days, did you do moderate activities for at least 10 minutes that cause only light sweating or a slight to moderate increase in breathing or heart rate? Some examples are brisk walking, bicycling for pleasure, golf, or dancing. (yes, no, unable to do activities)	PAD320	PAD320
	(IF YES) What activities did you do? code activity	PADACTIV	PADACTIV
	(IF YES) How often did you do X activity? enter number (past 30 days)	PADTIMES	PADTIMES
	(IF YES) On average, about how long did you do X each time? enter number of minutes	PADDURAT	PADDURAT
