# Supplementary material for: Association of physical activity with cardiovascular and renal outcomes and quality of life in chronic kidney disease
Source: PLoS One. 2017 Aug 23;12(8):e0183642. doi: 10.1371/journal.pone.0183642 (PMC5568323; doi:10.1371/journal.pone.0183642)
Supplement: S2 File — (PDF) [file pone.0183642.s002.pdf]

慢性腎臟病患者之體適能狀況評估之相關研究

體適能問卷

■**身體活動與運動量:(評估過去一個月身體活動與運動情形)**

身體活動量: (過去一個月中平均**每週**的運動時間，計算平均每週從事活動的時間)

**類別**	**運動項目**	**運動時間(每週總時間)**
□**費力身體活動量**:<br>心跳經常加快、運動時每週有3次↑心跳加快、自覺費力指數有點困難<br><br>做這些活動時會讓你沒辦法一邊活動，一邊跟別人輕鬆說話，這些活動會讓你身體感覺很累，呼吸會比平常快很多，流很多汗、心跳很快。任何活動強度跟”**跑步**”差不多就算。	□跑步<br>□爬山<br>□自行車 (16公里/小時↑)<br>□游泳(自由式)<br>□打網球<br>□打羽球<br>□其他：____________	■平均每週共______分鐘
□**中等費力身體活動**:<br>心跳偶爾加快、運動時每週有1-2次心跳加快、自覺費力指數輕鬆<br><br>做這些活動時還可以舒服的說話，但無法唱歌，這些活動會讓你身體有點累、有點喘、流一些汗、心跳快一些。任何活動強度跟”**快走**”差不多持續大於10分鐘就算。	□快走(5-6公里/小時)<br>□氣功<br>□種菜<br>□土風舞<br>□騎腳踏車<br>□慢泳<br>□勞力家事<br>□體操<br>□其他：____________	■平均每週共______分鐘
□**輕度身體活動量**:<br>心跳從不加快、運動時心跳無加快、自覺費力指數非常輕鬆不符合上述兩個標準的狀況	□散步<br>□其他：____________	■平均每週共______分鐘
□**無活動力**		

■個案身體活動量種類:
□高身體活動量(每週達60分鐘費力身體活動量）
□身體活動量足夠(每周達150分鐘中等費力身體活動量)
□身體活動量不足(不符合上述兩個標準的人)
□無活動力
